# Active control of dielectric singularities in indium-tin-oxides hyperbolic metamaterials

**DOI:** 10.1038/s41598-022-21252-x

**Published:** 2022-10-10

**Authors:** Alessandro Pianelli, Vincenzo Caligiuri, Michał Dudek, Rafał Kowerdziej, Urszula Chodorow, Karol Sielezin, Antonio De Luca, Roberto Caputo, Janusz Parka

**Affiliations:** 1 Institute of Applied Physics, Military University of Technology, 2 Kaliskiego Str., 00-908 Warsaw, Poland; 2 Faculty of Engineering and Natural Science, Photonics, Tampere University, 33720 Tampere, Finland; 3 Dipartimento di Fisica, Università della Calabria, 87036 Rende, Italy; 4 CNR Nanotec, Università della Calabria, 87036 Rende, Italy; 5 Institute of Fundamental and Frontier Sciences, University of Electronic Science and Technology of China, Chengdu, 610054 China

**Keywords:** Metamaterials, Nonlinear optics, Sub-wavelength optics

## Abstract

Dielectric singularities (DSs) constitute one of the most exotic features occurring in the effective permittivity of artificial multilayers called hyperbolic metamaterials (HMMs). Associated to DSs, a rich phenomenology arises that justifies the ever-increasing interest profuse by the photonic community in achieving an active control of their properties. As an example, the possibility to “canalize” light down to the nanoscale as well as the capability of HMMs to interact with quantum emitters, placed in their proximity, enhancing their emission rate (Purcell effect), are worth mentioning. HMMs, however, suffer of an intrinsic lack of tunability of its DSs. Several architectures have been proposed to overcome this limit and, among them, the use of graphene outstands. Graphene-based HMMs recently shown outstanding canalization capabilities achieving λ/1660 light collimation. Despite the exceptional performances promised by these structures, stacking graphene/oxide multilayers is still an experimental challenge, especially envisioning electrical gating of all the graphene layers. In this paper, we propose a valid alternative in which indium-tin-oxide (ITO) is used as an electrically tunable metal. Here we have numerically designed and analyzed an ITO/SiO_2_ based HMM with a tunable canalization wavelength within the range between 1.57 and 2.74 μm. The structure feature light confinement of λ/8.8 (resolution of about 178 nm), self-focusing of the light down to 0.26 μm and Purcell factor of approximately 700. The proposed HMM nanoarchitecture could be potentially used in many applications, such as ultra-fast signal processing, high harmonic generation, lab-on-a-chip nanodevices, bulk plasmonic waveguides in integrated photonic circuits and laser diode collimators.

## Introduction

Confining light at the nanoscale, below the diffraction limit, is one of the long-term challenges in the field of photonics. In this framework, artificial “metal”/dielectric multilayers—called hyperbolic metamaterials (HMMs)—emerged as one of the most promising solutions^1–5^. These materials feature exceptional anisotropy, captured in their effective uniaxial dielectric permittivity tensor, which is built as follows^6,7^:

1 $$\widehat{\varepsilon }=\left[\begin{array}{ccc}{\varepsilon }_{xx}& 0& 0\\ 0& {\varepsilon }_{yy}& 0\\ 0& 0& {\varepsilon }_{zz}\end{array}\right]$$

where, ${\varepsilon }_{xx}$= ${\varepsilon }_{yy}$=${\varepsilon }_{\parallel }$ are the in-plane components and ${\varepsilon }_{zz}$=${\varepsilon }_{\perp }$ is the out-of-plane component (optical axis). When the signs of ${\varepsilon }_{\parallel }$ and ${\varepsilon }_{\perp }$ are opposite, the resulting dispersion relation acquires the shape of a hyperboloid and the associated propagation regimes are called “Type I” (if ${\varepsilon }_{\parallel }>0$ and ${\varepsilon }_{\perp }<0$) or “Type II” (if ${\varepsilon }_{\parallel }<0$ and ${\varepsilon }_{\perp }>0$). Hyperbolic dispersions allow the HMMs to interact in unique ways with the electromagnetic radiation but when the Type I and Type II anisotropies, which are normally separated by an “effective dielectric” gap, touch each other, and reverse at one single point (the inversion point of coexisting anisotropies), a special condition occurs which is relevant for the purposes of this work. At the transition wavelength between the Type I and Type II dispersion regions, the HMM becomes capable to canalize light^8^ at a subwavelength scale. In other words, the light radiated at the transition wavelength by a dipole source placed in the vicinity of an HMM is canalized in a “soliton-like” straight beam, with a diameter comparable to and only limited by the periodicity of the HMM^9,10^. Canalized light propagating in such HMM shares many features with solitons, localized waves that propagate along one direction into a bulk or 2D media^11,12^.

**Figure 1:**
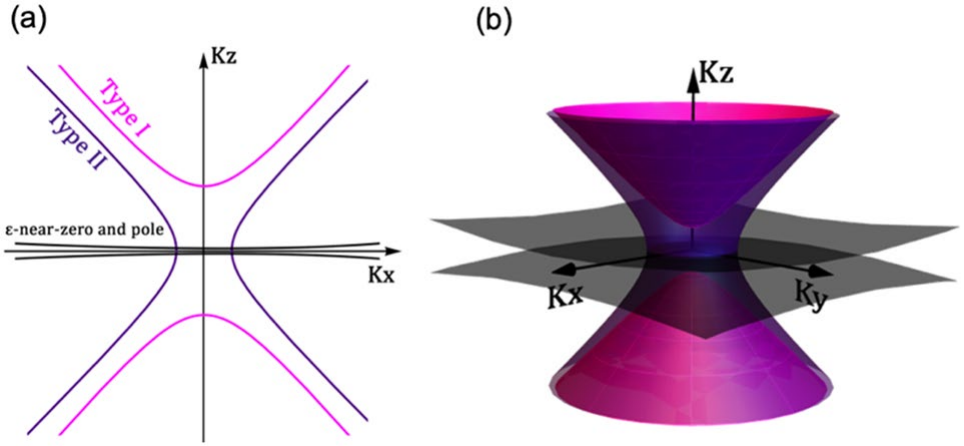
Sketch of 2D (**a**) and 3D (**b**) equi-frequency contour for Type I, Type II and ε-near-zero-and-pole regimes, respectively.

Neglecting ohmic losses and non-local effects, there are two constraints that make the occurrence of the canalization regime possible in HMMs^13^: (i) equal thickness of the metal and of the dielectric layer and (ii) real part of the dielectric permittivity of the two materials equal in modulus but opposite in sign. The canalization regime can be searched in the effective permittivity of the HMM. If the previous constraints are fulfilled, it turns that: (i) ${\varepsilon }_{\parallel }$ = ε_xx_ = ε_yy_ ≈ 0 (ε-near-zero) and (ii) ${\varepsilon }_{\perp }$ = ε_zz_ ≈ ∞ (ε-near-pole). Such extreme anisotropy, to which the canalization regime is associated, was previously called “ε-near-zero-and-pole” (ENZP)^14^. The achievement of the ENZP dispersion can be fully captured in the typical equi-frequency contour plots shown in Fig. 1a (2D plot) and Fig. 1b (3D plot). At the ENZP wavelength, the dispersion curves are almost flat in the XY plane meaning that very large wavevectors are allowed to propagate in the XY plane together with *k*_*z*_ values close to zero. The light propagates extremely slow in the XY plane “slow light effect”^15^ and extremely fast along the Z axis, thus eventually providing canalization of the electromagnetic field into the HMM.

Importantly, the Type I/Type II transition is not strictly necessary to achieve exceptional collimation of light, as recently demonstrated^16^. Indeed, if a singularity is still present in the ${\varepsilon }_{\perp }$, light confinement is still possible. Therefore, throughout this paper, we will refer to ENZP condition in this last, more general, way, without strictly implying a Type I/Type II transition.

Besides the typical ellipsometric characterization, another way to detect the occurrence of a hyperbolic regime, as well as the transition from the Type I to the Type II dispersion, is to exploit the capability of the HMM to enhance the decay rate of a fluorophore placed in its proximity^17,18^. Such a phenomenon, known as Purcell effect^19^, occurs in HMMs due to radiative interaction between fluorophores fostered by the enhancement of the photonic density of states manifested by HMMs in the hyperbolic region^20^. Such a relevant feature makes ENZP HMMs exceptional candidates as deep-subwavelength canalized single photon sources^21,22^.

However, there are several drawbacks that prevent the experimental realization of such technology. Among them, (i) the lack of design flexibility, (ii) losses and (iii) the absence of an active control of the canalization wavelength. Indeed, once a “metal/dielectric” pair has been selected to design an ENZP HMM, the canalization regime is attained at one precise wavelength, thus preventing the possibility of moving the canalization wavelength where desired.

Tentative solutions have been proposed to introduce a certain degree of design flexibility by adopting three-materials architectures^23^. To overcome losses at subwavelength scale the introduction of gain media has been proposed that counterpoise metallic losses^24,25^. In situ external control of the canalization wavelength has been numerically predicted in graphene-based HMMs^26^. Seminal investigations carried out by A. Boltasseva and H. A. Atwater^27^ envisioned a multitude of new materials such as Transparent Conductive Oxides (TCOs) that can be used in nanoscale photonics thanks to their plasmonic properties^28^ united to the possibility to achieve an active control of their optical constants^29,30^. ITO plays a special role^31–33^, due to its: (i) intrinsic ε-near-zero, (ii) low value of the negative permittivity (at the range of interest) and (iii) low losses. The most important characteristic that makes ITO perfect for the design we are going to showcase in this manuscript is the possibility to electrically tune its bulk plasma frequency in a broad range^34^. The intuition that fostered the research carried out here, is to numerically design a HMM with a broadband and electrically tunable ENZP wavelength, by using ITO as a low-loss gating metal-switch. By doing this, we demonstrate the possibility to achieve an active control of the ENZP topological regime from ~ 1.5 to ~ 2.7 μm associated to a fully reversible tunability of more than 1100 nm of the canalization regime. A super collimation effect and high resolution are reported in the focusing and self-focusing canalization regimes occurring at ENZP for each considered tunable wavelengths, achieving a resolution beyond the Abbe limit. We show an extreme focusing/self-focusing of light down to 0.26 μm and report a high value of the Purcell factor (PF) at each ENZP transition. We demonstrate that while electrically increasing the *N* number of carrier concentration in the ITO layer an enhancement of the canalization features of the HMM is achieved together with a decrease of the Purcell factor, thus imposing a trade-off between these two parameters. Moreover, we address an increase of the nonlinearity and the high-k modes^20^ (traced by topological parameter α^35^) at ε-near-zero transition as the *N* number of carrier concentration gets higher. The proposed structure is easy to fabricate via physical vapor deposition and, as such, can help overcoming the experimental limitation of graphene-based metamaterial architectures, holding great promise for applications such as ultrafast optical switch^36^, modulators^37^ and tunable HMM single-photon sources^21^.

## Results and discussion

**Figure 2:**
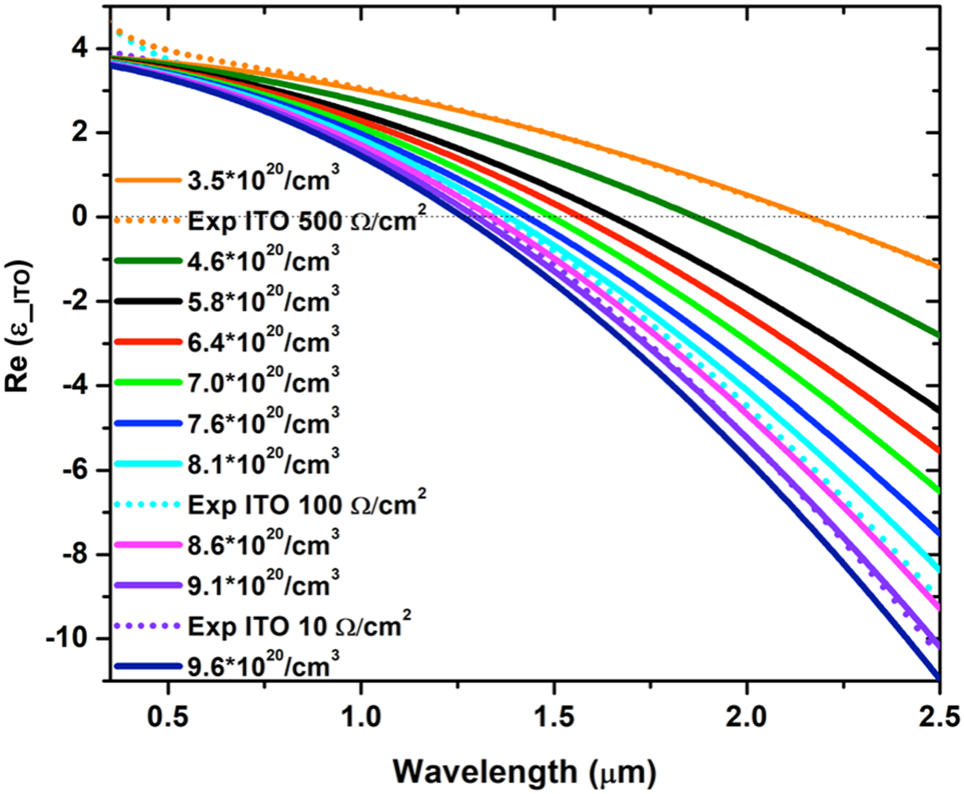
Tunable dielectric permittivity for different *N* carrier concentrations in the ITO layer.

The dielectric permittivity of ITO slab can be controlled via optical or electrical gating^29,38^. ITO-based HMMs also inherit such capabilities, as demonstrated here^39,40^. The application of an external potential or the interaction with a highly energetic optical pulse can change the carrier concentration “*N”* of the ITO and, consequently, its resistivity and plasma frequency. ITO resistivity can be directly tuned as well during the vapor deposition process by acting on parameters like substrate temperature and chamber pressure^33^. The effect is the same: a modification of the dielectric permittivity. In Fig. 2, the retrieved experimentally optical constants of three ITO layers with different permittivity (orange, cyan and purple dot curves) are compared with the ones modelled via simple Drude dispersion model (Supporting Information (SI) – section 1, 2, 3). Such scheme allows to retrieve the value of the free carriers of the ITO layers by simple comparison. Hence, for example, the sample with measured sheet resistance of 10 Ω/cm^2^ (purple dot curve) is characterized by a carrier concentration equal to $9.1\cdot{10}^{20}$/cm^3^.

**Figure 3:**
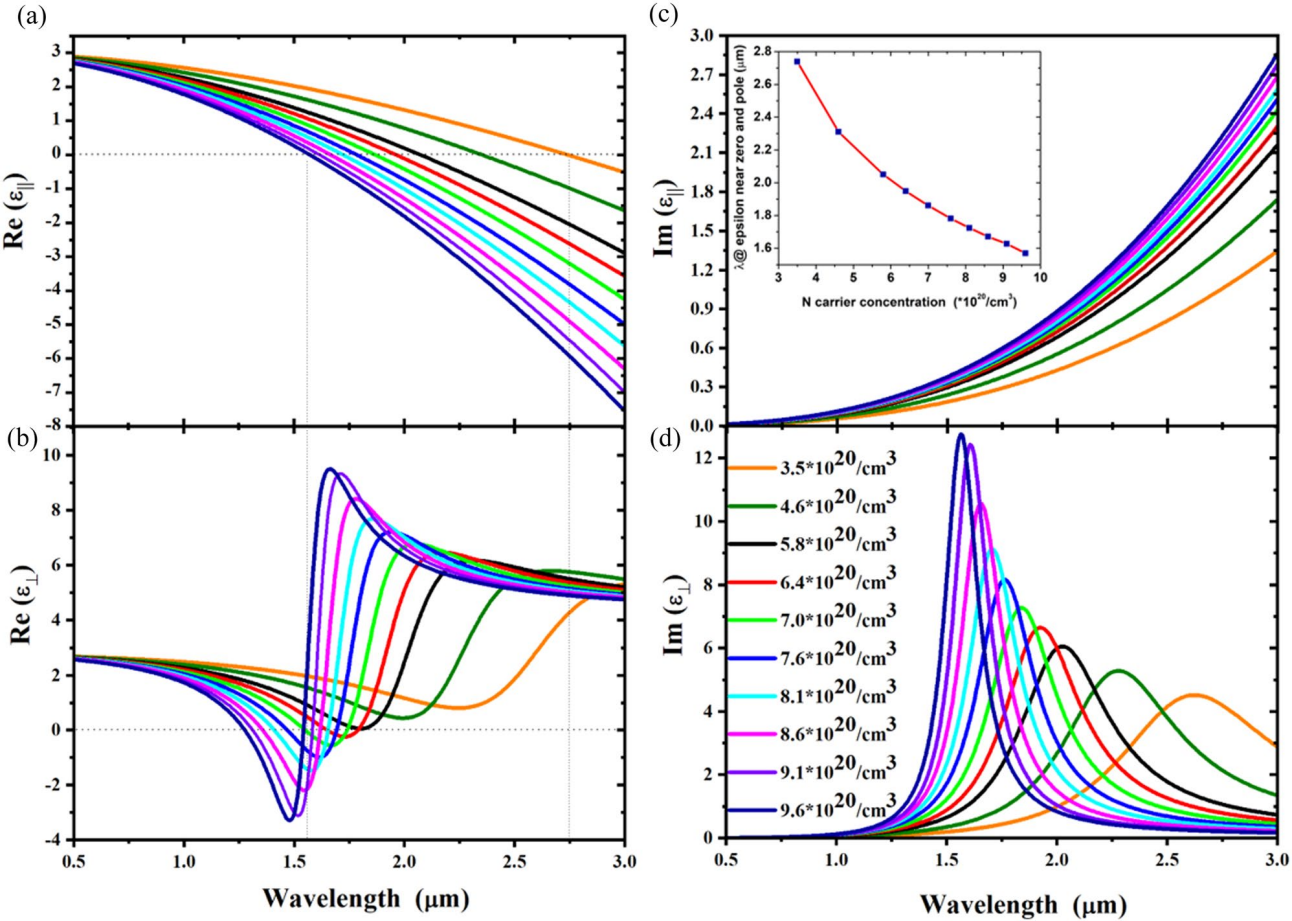
(**a**,**b**) Real part of parallel (${\varepsilon }_{\parallel }$) and perpendicular (${\varepsilon }_{\perp }$) effective dielectric permittivity modelled with effective medium theory (EMT) for a stack (21 layers) composed by 20 nm ITO (in function of *N* carrier concentration)—20 nm SiO_2_. (**c**,**d**) The imaginary part of the parallel (${\varepsilon }_{\parallel }$) and perpendicular (${\varepsilon }_{\perp }$) effective dielectric permittivity for the same stack, respectively. Inset in (**c**) shows each ENZP wavelengths versus *N* carrier concentration.

If the modification of the *N* carriers concentration is significant, the sign of the real part of the dielectric permittivity can flip, indicating the transition from the insulating to the metallic behaviour of the ITO layer, as shown in Fig. 2. Figure 2 also clarifies that, while increasing the number *N* of carriers concentration, the dielectric-to-metal transition in the ITO layer occurs at always shorter wavelengths, as expected. The broad tunability of the ITO *N* carriers concentration represents the key point to enable the same capability in the proposed HMM. Let us consider a “metal”/dielectric multilayer structured as 21 stacked layers in which 20 nm thick ITO represents the tunable metallic layer, whereas equal sized SiO_2_ is the passive dielectric. SiO_2_ is ideal for the purposes of this architecture due to its ease of deposition via physical or chemical vapor deposition techniques as well as for its wide bandgap that ensures perfect insulation between the stacked ITO layers. The broad tunability of the ITO dielectric permittivity allows to tailor the effective dielectric permittivity of the entire multilayer (HMM) in which the ITO is employed. In Fig. 3a,b the real parts of the in-plane (${\varepsilon }_{\parallel }$, Fig. 3a) and of the out-of-plane (${\varepsilon }_{\perp }$, Fig. 3b) components of the effective permittivity of the ITO/SiO_2_ HMM are shown for different *N* carriers concentrations.

For the investigated concentrations, the transition from positive to negative values of ${\varepsilon }_{\parallel }$ corresponds a flex in the ${\varepsilon }_{\perp }$ that, in correspondence to this wavelength, manifests the typical sigmoidal features of the anomalous dispersion in presence of a singularity. This wavelength represents the ENZP of the proposed HMM. While increasing *N*, the ENZP wavelength blue shifts, as shown in the inset of Fig. 3c. Accordingly, the imaginary part of the perpendicular (${\varepsilon }_{\perp }$) effective dielectric permittivity blueshifts, Fig. 3d, where a tunability range of more than 1100 nm is demonstrated passing from $N=3.5\cdot{10}^{20}$/cm^3^ to $N=9.6\cdot{10}^{20}$/cm^3^.

**Figure 4:**
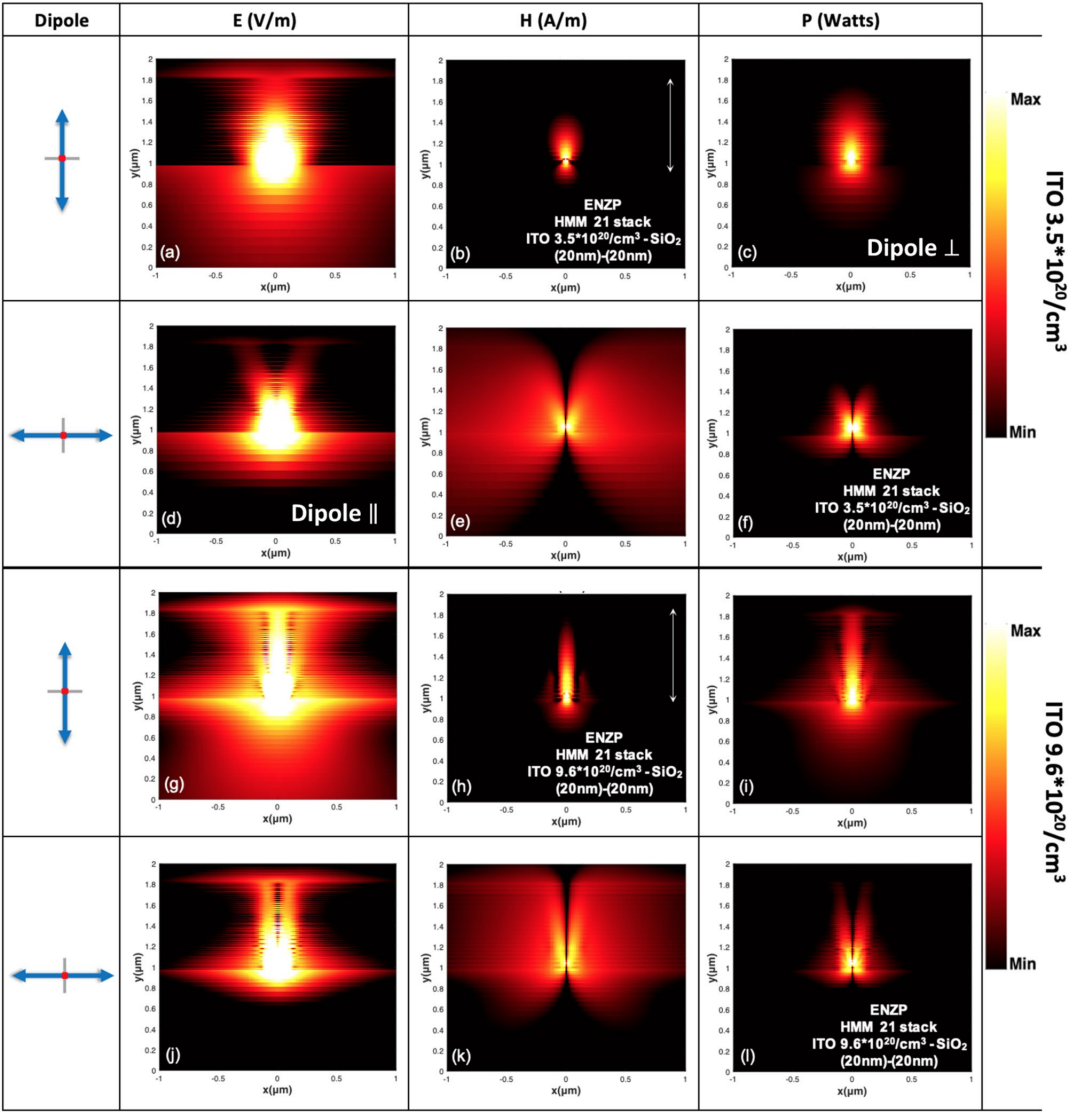
(**a**–**l**) Electric, magnetic and power near-field distributions (on a logarithmic scale) of perpendicular and parallel dipole source, placed in the bottom layer of the HMM, funneling the polaritons at ENZP regime for a 21 stack of HMM consisting of 20 nm ITO—20 nm SiO_2_, for modulated ITO *N* carrier concentration *N* = 3.5 * 10^20^/cm^3^ at λ = 2.74 μm and *N* = 9.6 * 10^20^/cm^3^ at λ = 1.57 μm, respectively.

The capability of the proposed HMMs to canalize light at each ENZP wavelength has been checked by means of FDTD simulations, where the excitation source is constituted by a dipole placed on the bottom layer of the HMM. The power distributions maps with those of the electric and magnetic field for both perpendicular and parallel dipole orientation (with respect to the HMM structure axes) are shown in Fig. 4a–l, for the two extreme cases of *N* = 3.5 · 10^20^/cm^3^ (upper panels) and *N* = 9.6 · 10^20^/cm^3^ (lower panels). They correspond to a shorter and longer canalization regime, respectively. (see the SI—section 4—Figure S4 to inspect the canalizations regime at intermediate *N* carrier concentrations).

The wavelengths at which ENZP singularity occurs in those two extreme cases are about 1.57 μm and 2.74 μm, respectively. A quick comparison between Fig. 4a,g demonstrates that canalization performance is better for the case of *N* = 9.6 · 10^20^/cm^3^. This is mainly due to the lower sheet resistance of the highly doped ITO layers that provides better out-of-plane free motion of electrons. However, the canalized light can propagate and spread into the structure, particularly for a large doping concentration. In fact, for higher sheet resistance for ITO 500 Ω/cm^2^ (*N* = 3.5 · 10^20^/cm^3^), we observed a shorter canalization regime. This is mainly due to the phase delay of the electromagnetic waves when they come in to contact with the HMM, as demonstrated in this work (see Figures S4 and S6—sections 4 and 5 in the SI). These considerations also find congruence in the calculated magnetic and power field profile, as can be concluded by comparing Fig. 4b,c (*N* = 3.5 · 10^20^/cm^3^), Fig. 4h,i (*N* = 9.6 · 10^20^/cm^3^) and for all the intermediate *N* carrier concentration (see the SI- section 4). For all the E, H and P distributions shown in the Fig. 4d–f,j–l, the light splits into a double branch in the HMM, with an angular dependence that gets narrower as the number of *N* carrier concentration increases. (see also SI—section 4, Figure S5). In Fig. 4e,k we show one of the capabilities of HMM that takes place at the DS: canalize and diffuse the light in the whole HMM, see the SI—section 4 (Figure S5) for the intermediated *N* number of the carrier concentration. This suggest that ENZP leads to the occurrence of a giant/control of the light-matter interaction that might enhance: spontaneous emission processes, photonic density of states and high second harmonic generation^41^.

### HMM as a tunable superlens

**Figure 5:**
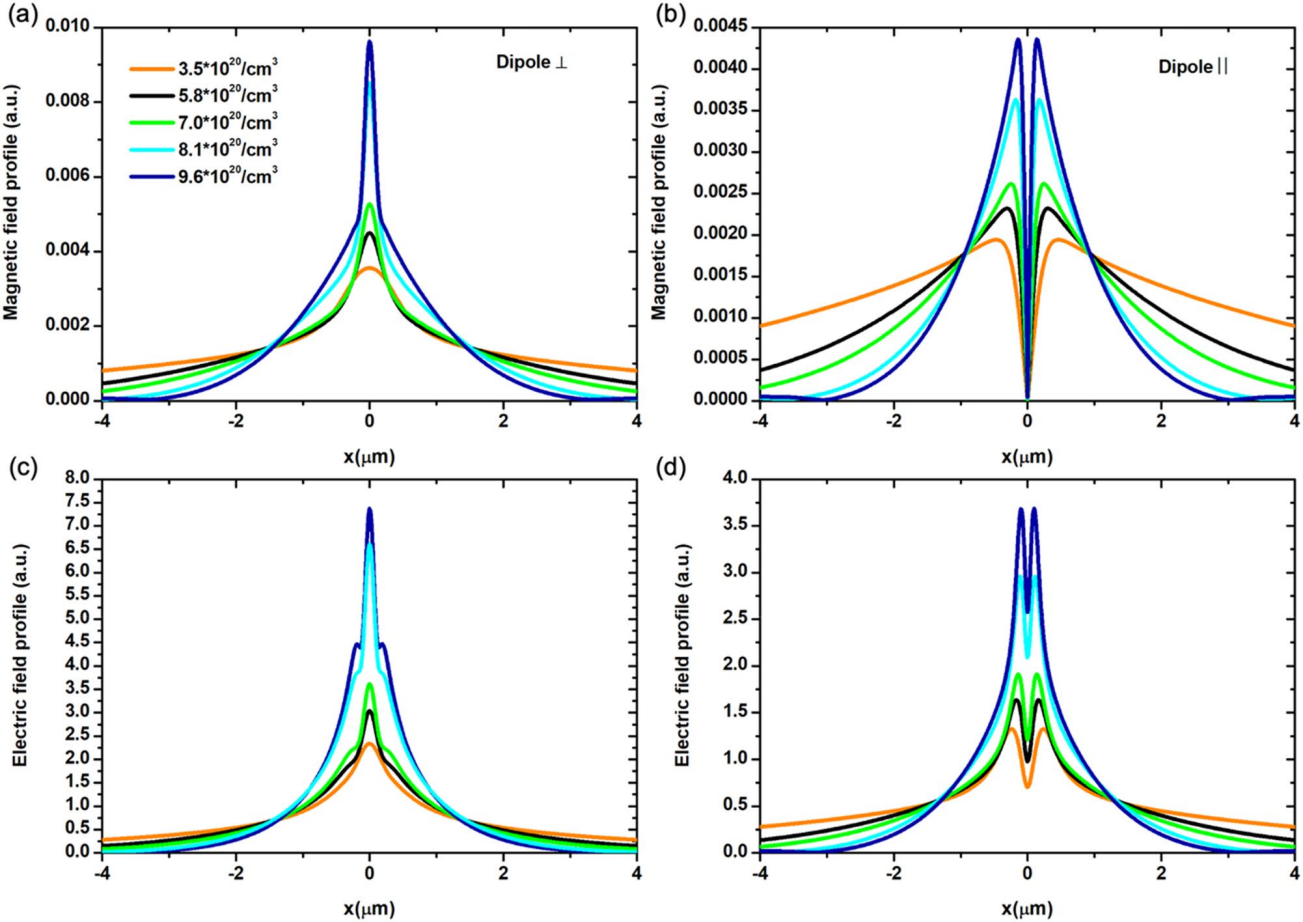
(**a**) Magnetic field profile for canalization regime at ENZP conditions, (**c**) (electric counterpart) focusing/self-focusing regime, (**b**,**d**) sub-wavelength resolution distribution for magnetic and electric field for a parallel dipole source placed in the bottom layer of the HMM.

A cut line at the exit of the HMM, highlights the subwavelength collimation and resolution capabilities of the proposed system at each ENZP wavelength as a function of *N* carrier concentration. In particular, the magnetic and electric field profile at the exit layer of the HMM for the perpendicularly oriented dipole are shown in Fig. 5a (magnetic) and Figure c (electric), while the same values for the parallel dipole are shown in Fig. 5b (magnetic) and Figure d (electric). A better canalization is observed for the magnetic (H) and electric (E) fields propagated into the HMM as soon as the *N* carrier concentration reaches the doping value of 9.6 · 10^20^/cm^3^ (blue curves in Fig. 5 a,d).

It is well known that the maximum resolution achievable via confocal microscopy can be calculated with the following formula^26,42^^:^

2 $$0.61\cdot\frac{\lambda n}{NA}$$

where $NA=1.4$ is the maximum obtainable numerical aperture of the objective and $n=1.5$ is the refractive index of the medium between the lens and the HMM (usually a matching index oil). The maximum resolution for the confocal microscope for each considered ENZP wavelengths and the corresponding Abbe diffraction limit are reported in the SI, Table S2—section 6. By placing the HMM structure into the microscope optical path, it would be possible to achieve a resolution down to $\lambda /8.8$ for an ENZP wavelength equal to 1569 nm and $\lambda /5.9$ for $\lambda =2739$ which are, respectively five and nearly fourfold resolution improvements far beyond the Abbe limit^43,44^.

For the sake of clarity, we summarized the subwavelength collimation capabilities in the following Tables 1 and 2 where the resolution was calculated for the two dipole orientation as *λ*_*ENZP*_*/D, D* being the focal spot in the self-focusing canalization regime and the sub-diffraction limited object peak-to-peak distance, respectively (see the focal spot data in that SI– section 6 Table S3, S4) of the beam propagating through the bulk of the HMM at the ENZP wavelengths:

**Table 1:** Hyperlens HMM at ε -near-zero and pole wavelengths: electric (E) and magnetic (H) counterpart for perpendicular dipole orientation.

ENZP working λ [nm]	(E) HMM collimation factor	(H) HMM collimation factor
λ = 2739	(λ/4.9)	(λ/2.9)
λ = 2067	(λ/4.0)	(λ/2.5)
λ = 1865	(λ/4.2)	(λ/3.6)
λ = 1724	(λ/4.8)	(λ/5.4)
λ = 1569	(λ/6.0)	(λ/5.6)

**Table 2:** Hyperlens HMM at ε -near-zero and pole wavelengths: electric (E) and magnetic (H) counterpart for parallel dipole orientation.

ENZP working λ [nm]	(E) HMM resolution	(H) HMM resolution
λ = 2739	(λ/5.9)	(λ/3.0)
λ = 2067	(λ/6.5)	(λ/3.6)
λ = 1865	(λ/7.2)	(λ/4.2)
λ = 1724	(λ/8.7)	(λ/5.4)
λ = 1569	(λ/8.8)	(λ/6.0)

Importantly, in our proposed metadevice, it would be possible to actively tune and switch at the wavelength of interest. Therefore, the HMM provides a wide range of high resolution at several wavelengths (1100 nm range) in the near-IR.

### Non-linearity, photonic density of states (PDOS) and Purcell effect engineering

**Figure 6:**
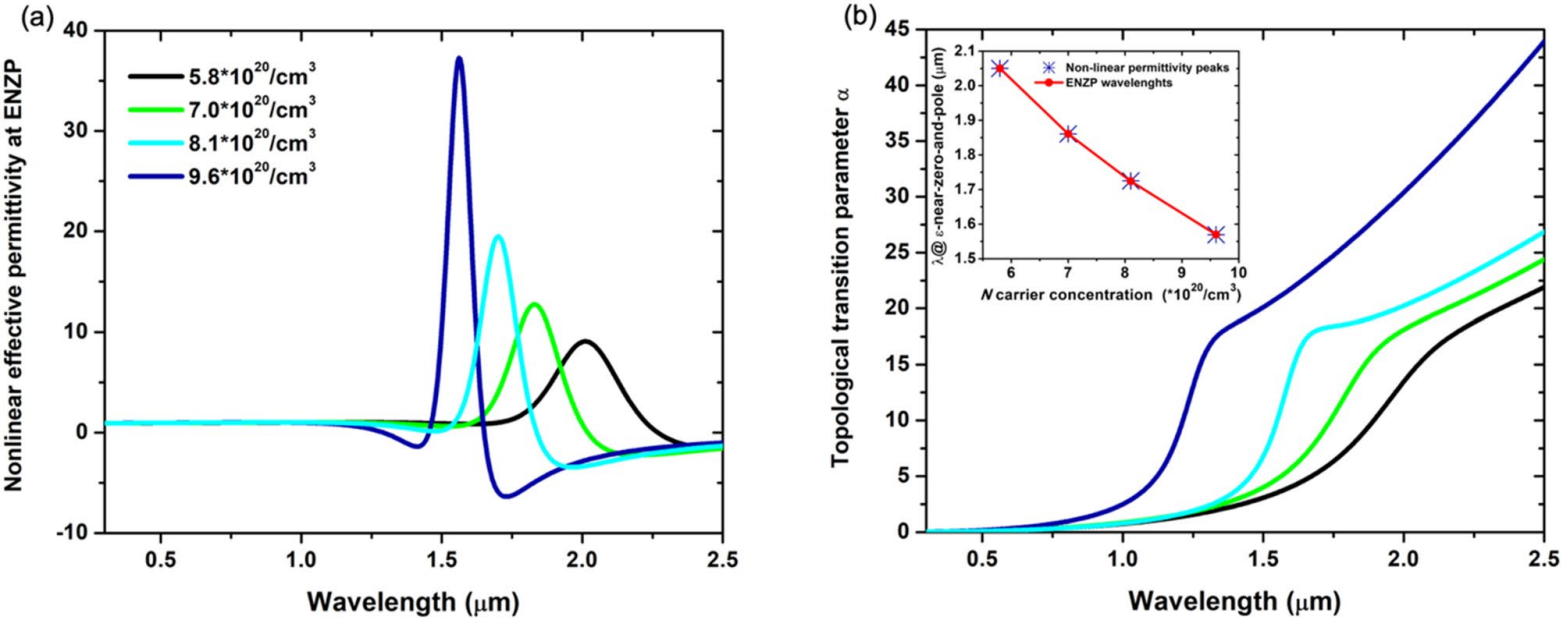
(**a**) Nonlinear effective permittivity at $\varepsilon$-near-zero-and-pole regimes and (**b**) topological transition parameter $\alpha$ for different *N* carrier concentration.

One of the most important possibilities enabled by HMM is that of engineering the photonic density of states (PDOS)^45^ in their proximity^46–50^. This feature makes HMMs exceptional for interaction with molecules or quantum confined fluorophores to enhance their decay rate through the well-known Purcell effect^17^. HMMs can manifest exceptionally high, eventually diverging, PDOS due to the large nonlinearities of their effective permittivities^51–53^. The non-linearity parameter at a precise wavelength can be retrieved as the ratio of the real parts of the in-plane ${\varepsilon }_{\parallel }$ and of the out-of-plane ${\varepsilon }_{\perp }$ components of the effective permittivity of the HMM and its value is shown in Fig. 6a as a function of the *N* free-carrier concentration of the ITO layers.

Strong nonlinear response of the considered HMM occurs in a very narrow range of wavelengths at which an extreme interchange of anisotropy takes place. The nonlinearity peaks occur in correspondence to the ENZP wavelengths. The highest nonlinearity is observed for the highest carrier concentration (*N* = 9.6 · 10^20^/cm^3^). Therefore, the topological transition parameter $\mathrm{\alpha }=\sqrt[2]{|{\varepsilon }_{\parallel }|{ \varepsilon }_{\perp }}/(1+|{\varepsilon }_{\parallel }|{ \varepsilon }_{\perp })$, proportional to the PDOS, gives a trace of the occurrence of high-k waves propagating within the HMM at each topological transition regimes^35^. In Fig. 6b, we reported the topological transition parameter α as a function of wavelength for different ITO *N* values. Importantly, α gets higher as the iso-frequency regime passes from Type I to Type II. A steeper response is observed at ENZP for ITO *N* = 9.6 · 10^20^/cm^3^, a sign of a remarkable increase of the high-k wave within the HMM. In the inset of the Fig. 6b, the peak value of the non-linear effective permittivity coincides with the ENZP wavelength for all the *N* carrier concentrations, confirming that strongest non-linearities occur at the ENZP wavelengths.

**Figure 7:**
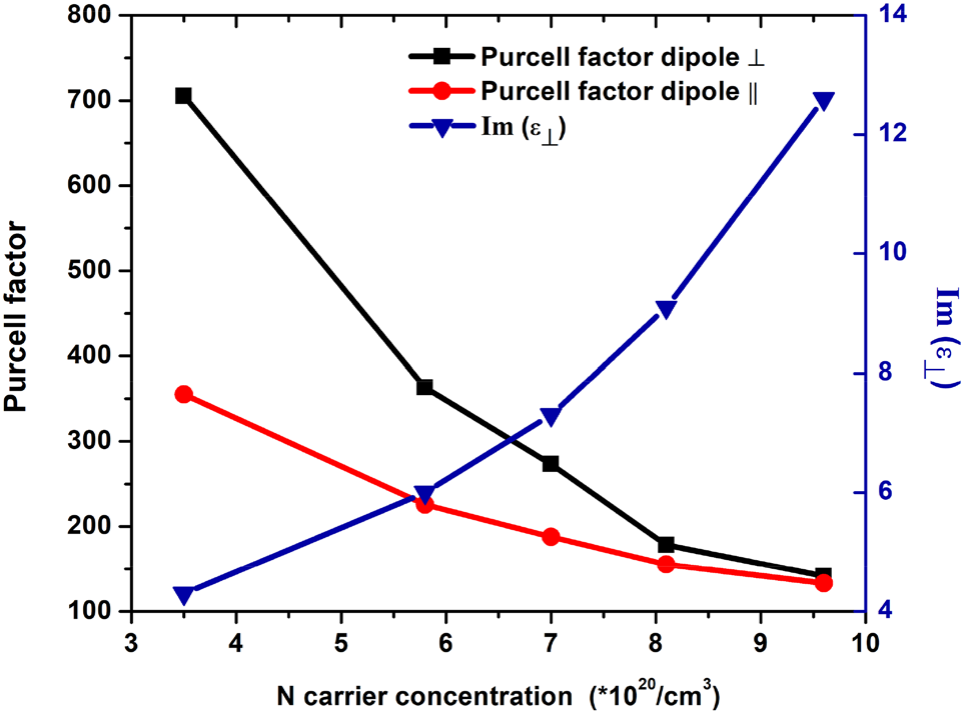
Purcell factor and Lorentzian imaginary part as a function of the *N* carrier concentration for a dipole placed in the bottom layer of the HMM, respectively parallelly and perpendicularly oriented.

Furthermore, nonlinearity enables the enhancement of PDOS, leading to a faster spontaneous emission process, particularly at ENZ^54^, which can be identified via Purcell factor (PF) modification. We calculated the PF for the proposed HMM architecture. We found that the Purcell enhancement highly depends on the *N* number of the carrier concentration. In fact, PF drops down nearly to sevenfold as the carrier concentration increases from 3.5 · 10^20^/cm^3^ to 9.6 · 10^20^/cm^3^, respectively (see Fig. 7).

Such a behavior can be understood considering that as the *N* carriers concentration increase, losses due to interband transitions increases accordingly. As a consequence, the presence of non-radiative processes in the HMM hinders the Purcell factor at each ENZP regimes. There is, therefore, a trade-off between high Purcell factor and subwavelength canalization. In fact, better canalization regime was reported here for large doping concentration. Hence, factors such as losses and resistivity, united with the Purcell effect must be considered in order to optimize the canalization regime.

## Conclusions

In this work we addressed the long-standing challenge of designing actively tunable ε-near-zero-and-pole hyperbolic metamaterials. The key to enable such a feature was the use of the ITO which, in the proposed architecture, plays the role of an electrically switchable metal. Leveraging, indeed, on the broad refractive index modification that can be induced in the ITO layer through electrical/optical gating, we demonstrated that the canalization wavelength of ITO/SiO_2_ HMM can be finely tuned in a broad range according to the electrical doping induced in the ITO layer. We demonstrated that, as expected, to an increase of *N* carriers concentration causes a better canalization effect, with a deep subwavelength confinement in the order of λ/8.8. We also showed that the strong nonlinearities associated to the dielectric singularities of the proposed HMMs lead to a strong Purcell factor at the ENZP wavelengths. The Purcell factor decreases as the *N* carriers concentration increases, imposing a trade-off between subwavelength collimation and Purcell factor. The proposed architecture is easily fabricable with respect, for example, to the graphene-based alternatives. As such, it holds great potential for ultra-fast electro/optical modulation, actively tunable deep-subwavelength imaging in the near-IR spectral region and enhance of the spontaneous emission process for single photon source.

## Material and method

In our approach, (ITO) slabs with sheet resistance of 10, 100, 500 Ω/cm^2^ respectively were characterized by ellipsometry measurements, retrieving the refractive index, extinction coefficient and the real and imaginary part of the permittivity via Drude-Lorentz and Tauc-Lorentz oscillator models^55^, see the supporting information (SI—section 1). We theoretically use an algorithm to fit the experimental optical constants to further tune the real part of the dielectric permittivity, see the demonstration in SI- section 2. Full-wave numerical simulations for ten distinct ITO *N* carrier concentration (see the Table 3) were performed, using ANSYS/Lumerical FDTD solutions software, for 21 stacks HMM composed by functional ITO 20 nm—SiO_2_ 20 nm (n = 1.45), see SI—section 3. SiO_2_ dielectric permittivity, equal to 2.1025, was modelled on the bases of Sellmeier’s expression valid in Vis–NIR range^56^.

**Table 3:** *N* ITO carrier concentration.

HMM ITO-SiO_2_	*N* carrier concentration [cm^−3^]
1. ITO	3.5·10^20^
2. ITO	4.6·10^20^
3. ITO	5.8·10^20^
4. ITO	6.4·10^20^
5. ITO	7.0·10^20^
6. ITO	7.6·10^20^
7. ITO	8.1·10^20^
8. ITO	8.6·10^20^
9. ITO	9.1·10^20^
10. ITO	9.6·10^20^

We designed the stack to parameterize the occurrence of the ε-near-zero-and-pole condition. In our method, the anisotropies in-plane (x–y) and out-of-plane (z) of the HMMs were determined employing effective medium theory (EMT)^6^

3 $${\varepsilon }_{\parallel }=\frac{{\varepsilon }_{{SiO}_{2}}^{{\prime}}{L}_{{SiO}_{2}}+ {\varepsilon }_{ITO}^{{\prime}}{ L}_{ITO}+ i({\varepsilon }_{{SiO}_{2}}^{{^{\prime\prime}}}{ L}_{{SiO}_{2}}+{\varepsilon }_{ITO}^{{^{\prime\prime}}} {L}_{ITO})}{{L}_{{SiO}_{2}}+ {L}_{ITO}}$$

4 $${\varepsilon }_{\perp }=\frac{{L}_{{SiO}_{2}}+ {L}_{ITO} }{{({\varepsilon }_{{SiO}_{2}}^{{\prime}} {L}_{ITO}+{\varepsilon }_{ITO}^{{\prime}}{ L}_{{SiO}_{2}})}^{2}+{({\varepsilon }_{{SiO}_{2}}^{{^{\prime\prime}}}{ L}_{ITO}+ {\varepsilon }_{ITO}^{{^{\prime\prime}}} {L}_{{SiO}_{2}})}^{2}} \{{\varepsilon }_{ITO}^{{\prime}}{ L}_{ITO} D+{\varepsilon }_{{SiO}_{2}}^{{\prime}}{L}_{{SiO}_{2}}M+i[{\varepsilon }_{ITO}^{{^{\prime\prime}}} {L}_{ITO}D+{\varepsilon }_{{SiO}_{2}}^{{^{\prime\prime}}}{ L}_{{SiO}_{2}}M]\}$$

where ${\varepsilon }^{{\prime}} and {\varepsilon }^{{^{\prime\prime}}}$ are the real and imaginary parts of the permittivity for both the building blocks used materials i.e., ITO and SiO_2_ with ${L}_{ITO}$ and ${L}_{{SiO}_{2}}$ thicknesses respectively. While $D={({\varepsilon }_{{SiO}_{2}}^{{\prime}})}^{2}+{({\varepsilon }_{{SiO}_{2}}^{{^{\prime\prime}}})}^{2}$ and $M={({\varepsilon }_{ITO}^{{\prime}})}^{2}+{({\varepsilon }_{ITO}^{{^{\prime\prime}}})}^{2}$.

The relations (3) and (4) reduces to the Eqs. (5) and (6) only in the case of low losses (i.e., the terms including ${\varepsilon }_{{SiO}_{2}}^{{^{\prime\prime}}}$ and ${\varepsilon }_{ITO}^{{^{\prime\prime}}}$):

5 $${\varepsilon }_{\parallel }=\frac{{\varepsilon }_{{SiO}_{2}}{L}_{{SiO}_{2}}+{\varepsilon }_{ITO}{ L}_{ITO}}{{L}_{{SiO}_{2}}+ {L}_{ITO}}$$

6 $${\varepsilon }_{\perp }=\frac{{\varepsilon }_{{SiO}_{2}}{\varepsilon }_{ITO}{ (L}_{ITO}+ {L}_{{SiO}_{2}})}{{L}_{{SiO}_{2}}{\varepsilon }_{ITO}+{L}_{ITO}{\varepsilon }_{{SiO}_{2}}}$$

The ε-near-zero-and-pole effect takes place if and only if the numerator and denominator of the relations (5) and (6) gets simultaneously zero. Essentially, this scenario can be constructed via having materials whose dielectric permittivity values result to be equal in magnitude at certain specific wavelength (with opposite sign) and equal thicknesses, respectively: ${\varepsilon }_{ITO}^{{\prime}}=-{\varepsilon }_{{SiO}_{2}}^{{\prime}}$ and ${L}_{ITO}={L}_{{SiO}_{2}}$. To analyse the electromagnetic field and the power distribution inside the structures, we utilize an oscillating dipole placed in the bottom layer of the HMM such that it acts as a source of electromagnetic waves that illuminates the HMM under consideration. To ensure simulations stability, the dipole source has been placed in the first lossless layer i.e., SiO_2_, so that the entire dipole injection mesh result to be within the first unit cell. Additionally, to assure consistency of the algorithm, a high mesh accuracy of numerical simulations was settled. Moreover, we used PML boundary conditions surrounding the whole structure, uniform spatial grid with 100 nm step and additional conformal mesh around the HMM structure with 10 nm step. The emission rate enhancement of spontaneous emission: Purcell factor, is retrieved based on FDTD simulation as the ratio of the dipole power radiated in the vicinity of the HMM and the source power radiated by the dipole in a homogeneous environment. Worth to mention is that the dipole injection source simulations, in ANSYS/Lumerical FDTD solutions, are solved and in full agreement with the Green’s function formalism^57–59^ has demonstrated here^60^.

## Supplementary Information

Supplementary Information.

## Data Availability

The datasets generated and analyzed during the current study are available from the corresponding authors on reasonable request.
