# Supplementary material for: Active control of dielectric singularities in indium-tin-oxides hyperbolic metamaterials
Source: Sci Rep. 2022 Oct 10;12:16961. doi: 10.1038/s41598-022-21252-x (PMC9551065; doi:10.1038/s41598-022-21252-x)
Supplement: Supplementary file 1 — Supplementary Information. [file 41598_2022_21252_MOESM1_ESM.docx]

**Supporting information**

**Active Control of Dielectric Singularities in Indium-Tin-Oxides hyperbolic metamaterials**

*Alessandro Pianelli*^§∂^**, Vincenzo Caligiuri^†Ϟ^*,Michał Dudek*^§^**, Rafał Kowerdziej*^§^*,* Urszula Chodorow^§^*, Karol Sielezin*^§^*, Antonio De luca^†Ϟ^, Roberto Caputo*^†¥^*, Janusz Parka*^§^

^§^ Institute of Applied Physics, Military University of Technology, 2 Kaliskiego Str., 00-908 Warsaw, Poland

^∂^Faculty of Engineering and Natural Science, Photonics, Tampere University, Tampere 33720, Finland

^†^Dipartimento di Fisica, Università della Calabria, 87036 Rende, Italy.

*^Ϟ^*CNR Nanotec, Università della Calabria, 87036 Rende, Italy.

^¥^Institute of Fundamental and Frontier Sciences, University of Electronic Science and Technology of China, Chengdu 610054, China

*****Corresponding authors:** [alessandro.pianelli@wat.edu.pl](mailto:alessandro.pianelli@wat.edu.pl); [Vincenzo.caligiuri@unical.it](mailto:Vincenzo.caligiuri@unical.it); [michal.dudek@wat.edu.pl](mailto:michal.dudek@wat.edu.pl).

**Contents**

1. **Experimental extraction of optical constant of ITO slabs**
2. **Model to tune the dielectric permittivity of ITO**
3. **Active modulation of ITO permittivity**
4. **Canalization regime at ENZP for different *N* number of the carrier concentration**
5. **Phase of the HMM in function of the *N* number of the carrier concentration at ENZP regime**
6. **HMM as a tunable superlens resolution data**
7. **Experimental extraction of optical constant of ITO slabs**

Samples under investigation were commercially available ITO films. We have used three different ITO slabs, shown in the **Figure S1**, deposited on float glass substrate with thickness of 1.1.mm:

| Samples | Sheet resistance (Ω/cm^2^) | Thickness + roughness (nm) | Mean square error of Psi & Delta fitting (MSE) | Oscillator model |
| --- | --- | --- | --- | --- |
| 1. ITO | 10 | 180+5 nm | 4.76 | Drude-Lorentz+Tauc-Lorentz |
| 2. ITO | 100 | 20+3 nm | 1.04 | Drude-Lorentz |
| 3. ITO | 500 | 12+6nm | 0.80 | Drude-Lorentz+Tauc-Lorentz |

Each ITO slab was characterized via spectroscopic ellipsometry to retrieve optical constants. Ellipsometric data were taken via using a Sentech SE 850 ellipsometer for angles from 60° to 70° by step increments of 5°. Fitting was performed with the SpectraRay/4 software. Here below, in the Figure S2, we report the refractive index, extinction coefficient and the complex dielectric permittivity retrieved via Drude oscillator model [1] for different ITO samples resistance of 10, 100 and 500 Ω/cm^2^, respectively. Refractive indices and extinction coefficients were used to perform further simulations employing the Effective Medium Theory (EMT) over the designed HMM.
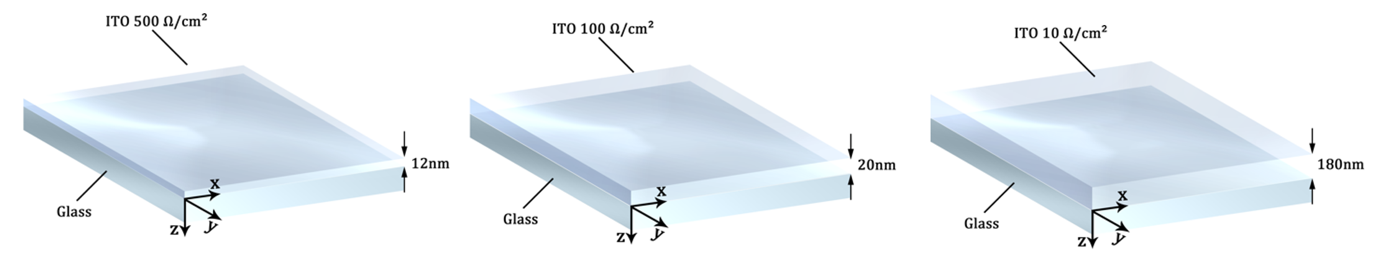


**Figure S1:** Sketch of the investigated ITO slabs, from the left to the right: ITO 500, 100, 10 Ω/cm^2^.


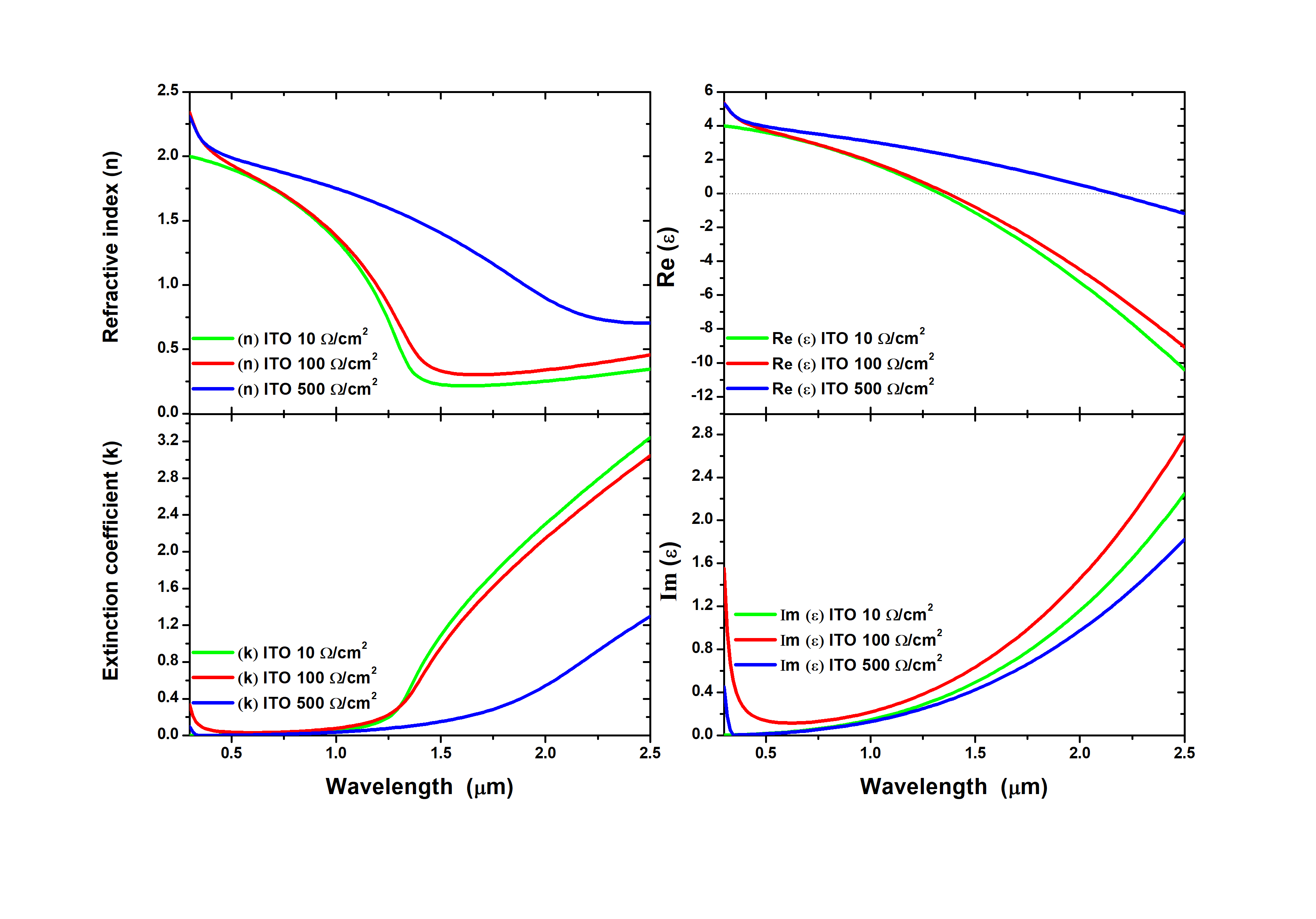


**Figure S2:** Refractive index (n), extinction coefficient (k) and real and imaginary parts of the ITO permittivity with sheet resistance equal to 10, 100, 500 Ω/cm^2^, respectively.

1. **Model to tune the dielectric permittivity of ITO**

We assessed the agreement between the complex permittivity, obtained from ellipsometric measurements, with a standard Drude analytical model to ensure the reliability of tunning/modulation of the effective dielectric permittivity of the HMM. In the **Figure S3** is reported the simulated (circle curve) and experimental permittivity (solid curve) for three different ITO slabs. Accordingly, the results indicate agreement between raw experimental data and the analytical Drude fit for the real and imaginary part of the dielectric permittivity, respectively. In particular, the fit is in full agreement at the epsilon-near-zero regime respectively for each considered ITO slab.


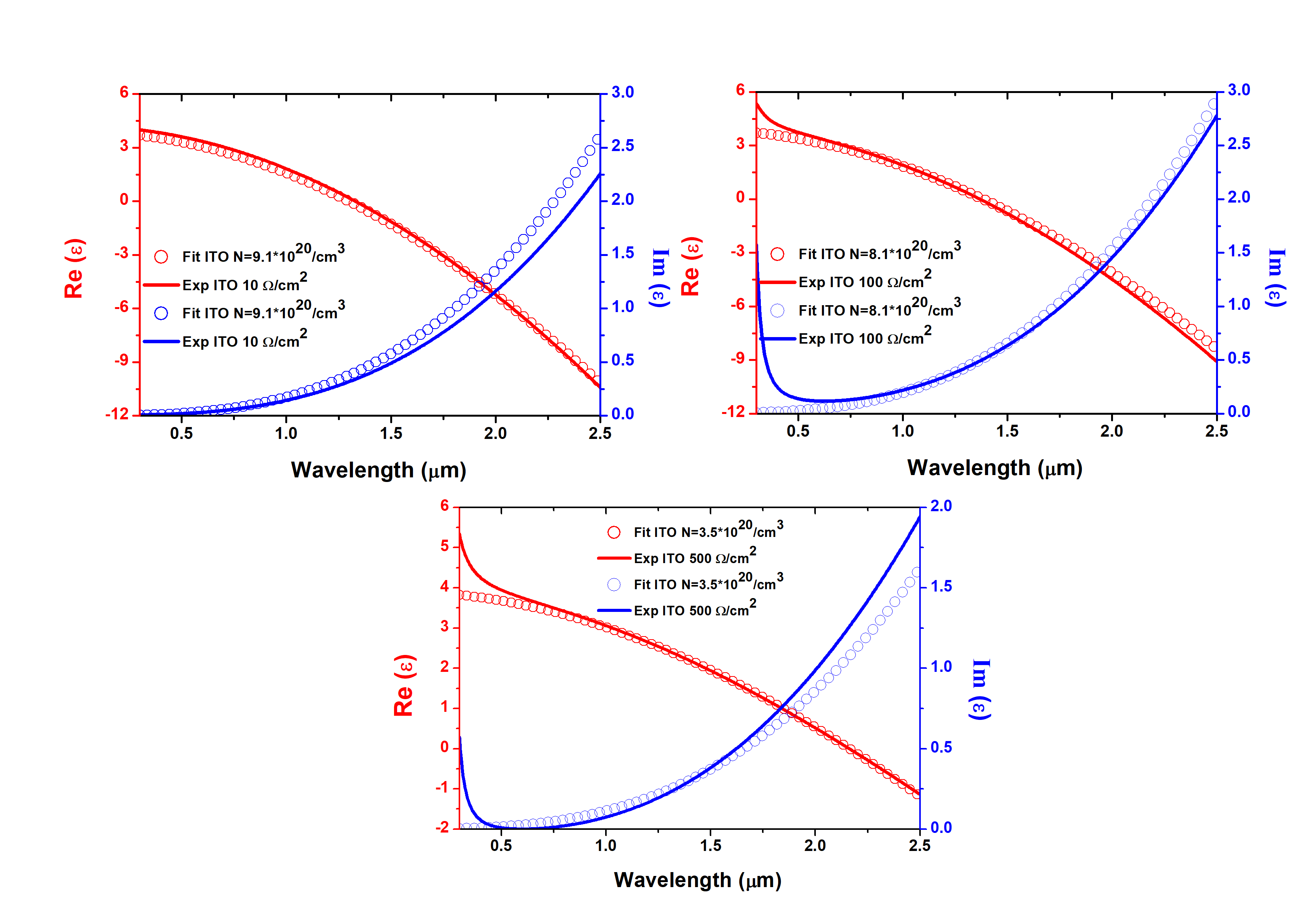


**Figure S3:** Relation between experimental and modelled ITO permittivity respectively for: ITO 10 Ω/cm^2^, 100 Ω/cm^2^ and 500 Ω/cm^2^.

Here, in the table below, a summary of pivotal material parameters:

**Table S1.**

| Samples | Zero permittivity condition Re(ε)=0 [μm] | Damping constant Γ [radHz] | High frequency permittivity $\boldsymbol{\varepsilon}_{\boldsymbol{\infty}}$ | *N* carrier concentration [cm^-3^] | Plasma frequency [Hz] | Carrier mobility [cm^2^/Vs] |
| --- | --- | --- | --- | --- | --- | --- |
| ITO 10 Ω/cm^2^ | 1.29317 μm | 1.4**⋅**10^14^ | 3.9 | 9.1**⋅**10^20^ | 1.45⋅10^15^ | 35.89 |
| ITO 100 Ω/cm^2^ | 1.37067 μm | 1.8**⋅**10^14^ | 3.9 | 8.1**⋅**10^20^ | 1.37⋅10^15^ | 27.92 |
| ITO 500 Ω/cm^2^ | 1.92967 μm | 2.4**⋅**10^14^ | 3.9 | 3.5**⋅**10^20^ | 0.90⋅10^15^ | 20.94 |

1. **Active modulation of ITO permittivity**

The ITO permittivity can be tuned and described in the Vis-near IR range via using the Drude model^2-10:^

$$\varepsilon_{ITO}\left( \omega\right)=\varepsilon_{\infty}-\left( \frac{\omega_{p}^{2}}{\left( \omega^{2}+i\Gamma\omega\right)} \right), \omega_{p}^{2}=\frac{Ne^{2}}{\varepsilon_{0}m_{eff}}$$

where $\omega_{p}$ is the plasma frequency, $m_{eff}=0.35{\cdot m}_{e},$ is the effective mass and $m_{e}$ is the electron rest mass. Here $\varepsilon_{0}$ is the dielectric permittivity of vacuum, $e$ is the electron charge. The following parameters were used to tune the permittivity of the ITO for different *N* carrier concentration$. \varepsilon_{\infty,}$ which is the high-frequency dielectric permittivity, and Γ, which is the damping constant (electron scattering frequency), all in agreement with the experimental data reported in Table 1. Since we consider a sub-wavelength unit cell composed by ITO 20 nm and SiO_2_ 20 nm respectively in the stack structure, the effective response of the HMM can be analyzed by the EMT^11^ which is widely used method to generalize the effective medium approximation excluding potential occurring diamagnetic effects^12^.

Two important factors are worth to mention:

1. Experimentally the ITO can be heavily doped by RF magnetron sputtering, at which the carrier concentration can be controlled by changing the ratio of the Ar and O_2_ flow rates, with the possibility to achieve a two-fold optical magnitudes orders more via gating.
2. The dielectric counterpart has to be carefully chosen to ensure the presence of high-k dielectric quality that sustain eventually high carrier concentrations in the ITO layers^2^.
3. **Canalization regime at ENZP for different *N* number of the carrier concentration**

We report, in **Figures S4** and **S5**, the distribution plots of the electric, magnetic and power for intermediate *N* number of the carrier concentration from 5.8*10^20^/cm^3^ to 9.1*10^20^/cm^3^ (jumping one concentration) respectively for dipole placed perpendicular and parallel in the bottom layer of the HMM.


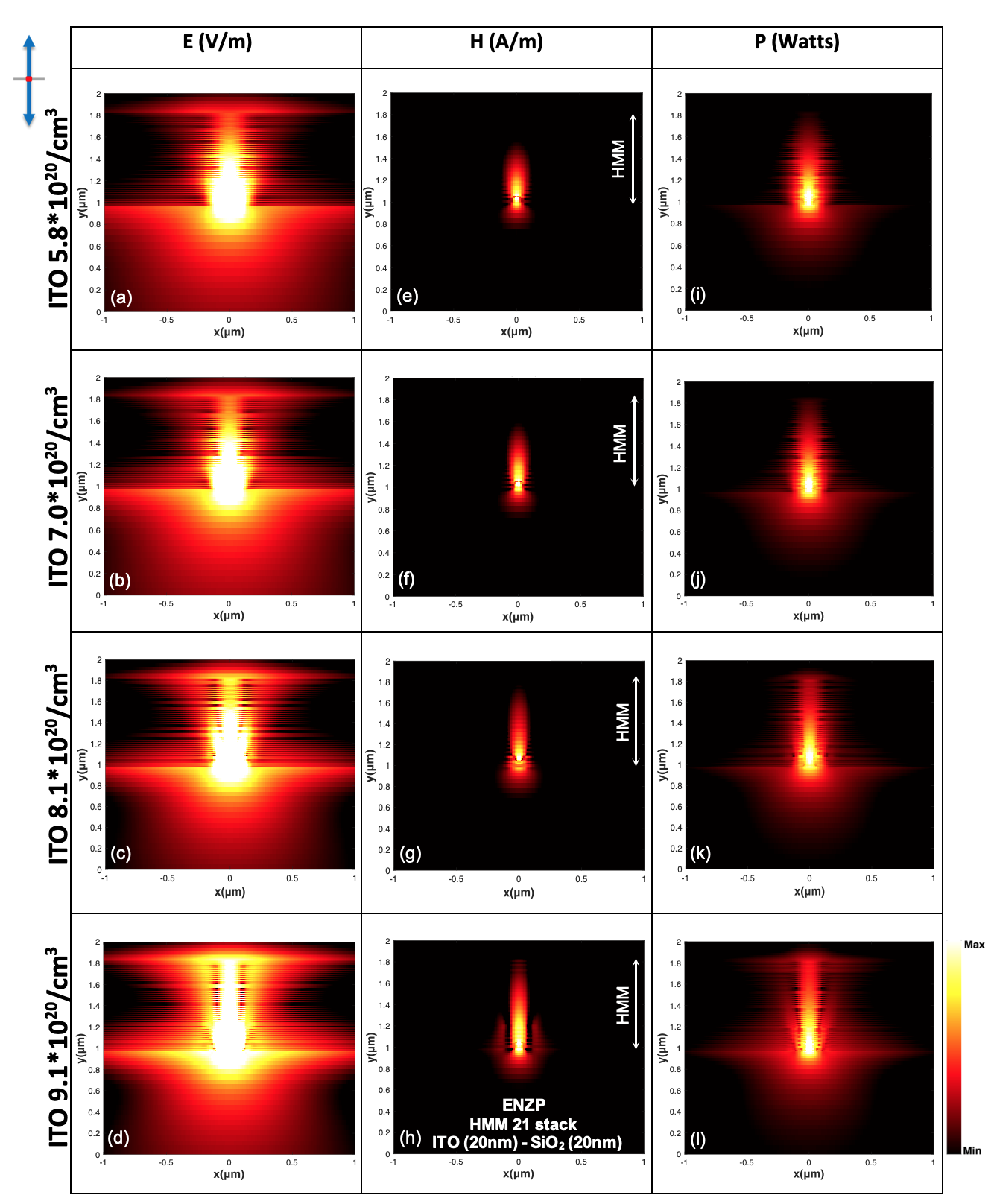


**Figure S4:** (a-d) Electric, (e-h) magnetic and power (i-l) near-field distributions (on a logarithmic scale) of perpendicular dipole source, placed in the bottom layer of the HMM, funneling the polaritons at ENZP regime for a 21-layer stack of HMM consisting of 20 nm ITO – 20 nm SiO_2_, for modulated ITO carrier concentration: 5.8*10^20^/cm^3^, 7.0*10^20^/cm^3^, 8.1*10^20^/cm^3^, 9.1*10^20^/cm^3^, respectively.

As can be seen, the electromagnetic field, injected into the HMM via the dipole at the specific ENZP wavelength, gets higher collimated in the whole HMM as the *N* carrier concentration increases (**Figure S4**). Furthermore, the double branch VPPs at ENZP gets narrower as the carrier concentration increases (**Figure S5**).


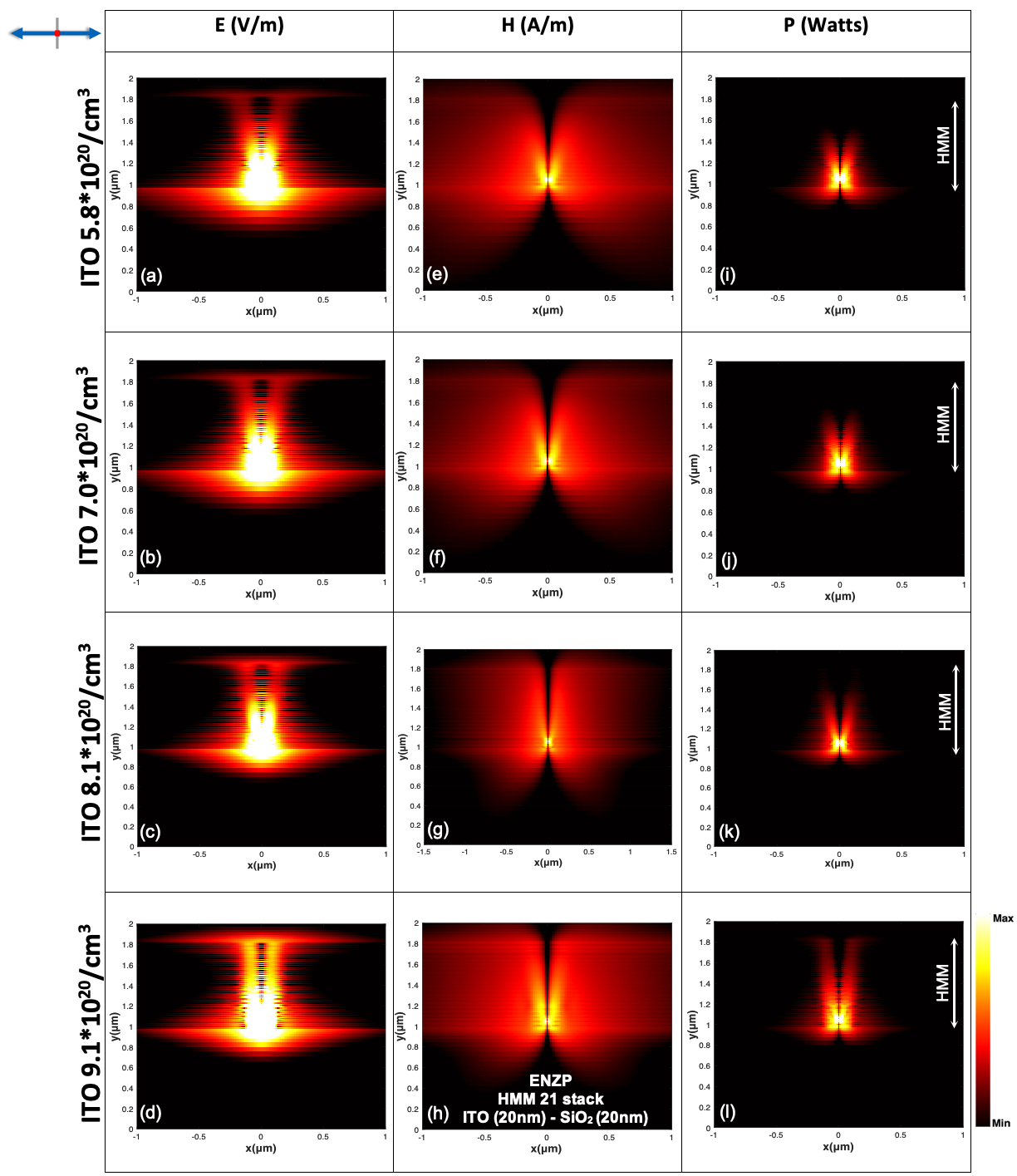


**Figure S5:** (a-d) Electric, (e-h) magnetic and power (i-l) near-field distributions (on a logarithmic scale) of parallel dipole source, placed in the bottom layer of the HMM, funneling the polaritons at ENZP regime for a 21-layer stack of HMM consisting of 20 nm ITO – 20 nm SiO_2_, for modulated ITO carrier concentration: 5.8*10^20^/cm^3^, 7.0*10^20^/cm^3^, 8.1*10^20^/cm^3^, 9.1*10^20^/cm^3^, respectively.

1. **Phase of the HMM in function of the *N* number of the carrier concentration at ENZP regime**

In the **Figure S6**, presented below, it is possible to observe the change in phase of the E, H, P into the HMM as a function of the *N* carrier concentration. As the *N* number of the carrier gets higher a better phase-match interplay between the HMM and the incoming dipole frequency oscillations is observed. Thus, a better collimation of light, within the structure, takes place due to the nearly in-phase interaction between the dipole and the HMM.


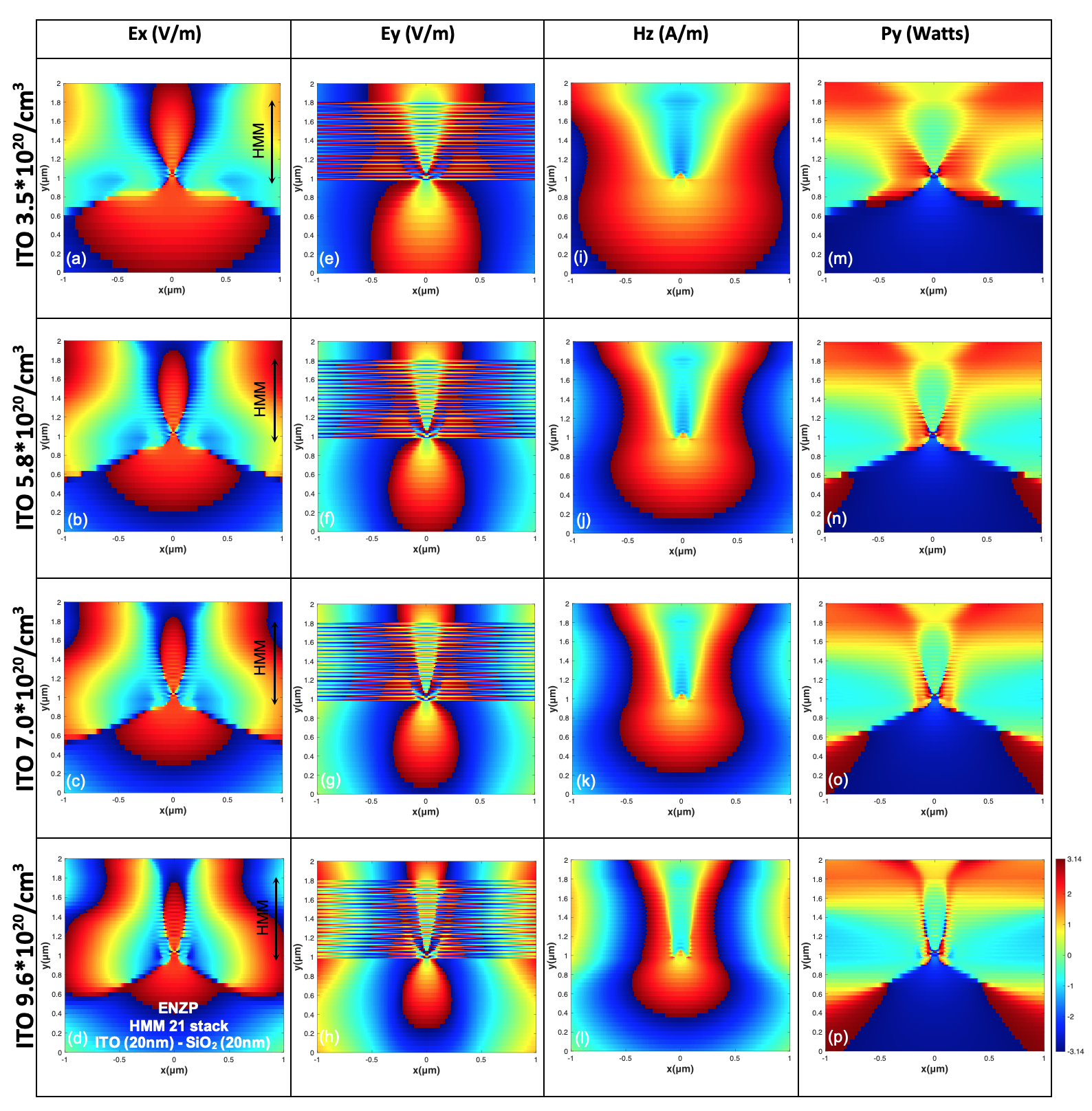


**Figure S6:** (a-d)(e-h) Electric filed phase response on the x and y axis, (i-l) Magnetic filed phase response on z axis and (m-p) power field phase response on y axis (in radiant units, respectively) for a dipole placed in the bottom layer of the HMM, funneling polaritons- at ENZP regime, for modulated ITO carrier concentration: 5.8*10^20^/cm^3^, 7.0*10^20^/cm^3^, 8.1*10^20^/cm^3^, 9.1*10^20^/cm^3^, respectively.

1. **HMM as a tunable superlens resolution data**

**Table S2.** **Hyperlens HMM at *ε -near-zero and pole wavelengths: max resolution and Abbe limit***

| ENZP working λ [nm] | Max resolution for confocal microscope (λ/1.53) | Abbe limit ($\boldsymbol{\lambda}$/2.8) |
| --- | --- | --- |
| λ= 2739 | 1790 nm | 978 nm |
| λ= 2067 | 1350 nm | 738 nm |
| λ= 1865 | 1218 nm | 666 nm |
| λ= 1724 | 1724 nm | 615 nm |
| λ= 1569 | 1025 nm | 560 nm |

**Table S3.** **Hyperlens HMM at *ε -near-zero and pole wavelengths: electric (E) & magnetic (H) counterpart for perpendicular dipole orientation***

| ENZP working λ [nm] | (E) subwavelength collimation focal spot  [nm] | (H) subwavelength collimation focal spot [nm] |
| --- | --- | --- |
| λ= 2739 | 560 | 940 |
| λ= 2067 | 520 | 820 |
| λ= 1865 | 440 | 520 |
| λ= 1724 | 360 | 320 |
| λ= 1569 | 260 | 280 |

**Table S4.** **Hyperlens HMM at *ε -near-zero and pole wavelengths: electric (E) & magnetic (H) counterpart for parallel dipole orientation***

| ENZP working λ [nm] | (E) Sub-diffraction limited object peak-to-peak distance [nm] | (H) Sub-diffraction limited object peak-to-peak distance [nm] |
| --- | --- | --- |
| λ= 2739 | 460 | 920 |
| λ= 2067 | 320 | 580 |
| λ= 1865 | 260 | 440 |
| λ= 1724 | 198 | 320 |
| λ= 1569 | 178 | 260 |

**References**

1. Maier, S. A. *Plasmonics: fundamentals and applications* (Vol. 1, p. 245). New York: springer 2007.

2. Papadakis, G. T., & Atwater, H. A. Field-effect induced tunability in hyperbolic metamaterials. *Physical Review B* 2015, **92**(18), 184101.

3. Alam, M. Z., De Leon, I., & Boyd, R. W. Large optical nonlinearity of indium tin oxide in its epsilon-near-zero region. *Science* 2016, **352**(6287), 795-797.

4. Liu, X., Park, J., Kang, J. H., Yuan, H., Cui, Y., Hwang, H. Y., & Brongersma, M. L. Quantification and impact of nonparabolicity of the conduction band of indium tin oxide on its plasmonic properties. *Applied Physics Letters* 2014, **105**(18), 181117.

5. Feigenbaum, E., Diest, K., & Atwater, H. A. Unity-order index change in transparent conducting oxides at visible frequencies. *Nano letters* 2010, **10**(6), 2111-2116.

6. Lee, Ho W., Georgia Papadakis, Stanley P. Burgos, Krishnan Chander, Arian Kriesch, Ragip Pala, Ulf Peschel, and Harry A. Atwater. "Nanoscale conducting oxide PlasMOStor." *Nano letters* 2014, **14**, no. 11 : 6463-6468.

7. Yi, F., Shim, E., Zhu, A. Y., Zhu, H., Reed, J. C., & Cubukcu, E. Voltage tuning of plasmonic absorbers by indium tin oxide. *Applied Physics Letters* 2013, **102**(22), 221102.

8. Vasudev, A. P., Kang, J. H., Park, J., Liu, X., & Brongersma, M. L. Electro-optical modulation of a silicon waveguide with an “epsilon-near-zero” material. *Optics express* 2013, **21**(22), 26387-26397.

9. Michelotti, F., Dominici, L., Descrovi, E., Danz, N., & Menchini, F. Thickness dependence of surface plasmon polariton dispersion in transparent conducting oxide films at 1.55 μm. *Optics letters* 2009, **34**(6), 839-841.

10. Huang, Y. W., Lee, H. W. H., Sokhoyan, R., Pala, R. A., Thyagarajan, K., Han, S., ... & Atwater, H. A. Gate-tunable conducting oxide metasurfaces. *Nano letters* 2016, **16**(9), 5319-5325.

11. Agranovich, V. M., & Kravtsov, V. E. Notes on crystal optics of superlattices. *Solid State Communications* 1985, **55**(1), 85-90.

12. Papadakis, G. T., Yeh, P., & Atwater, H. A. Retrieval of material parameters for uniaxial metamaterials. *Physical Review B* 2015, **91**(15), 155406.
